# Effects of adding *Allium mongolicum* Regel powder and yeast cultures to diet on rumen microbial flora of Tibetan sheep (*Ovis aries*)

**DOI:** 10.3389/fvets.2024.1283437

**Published:** 2024-02-21

**Authors:** Chunhui Wang, Juan Fan, Keyan Ma, Huihui Wang, Dengpan Li, Taotao Li, Youji Ma

**Affiliations:** ^1^College of Animal Science and Technology, Gansu Agricultural University, Lanzhou, China; ^2^Gansu Key Laboratory of Animal Generational Physiology and Reproductive Regulation, Lanzhou, China

**Keywords:** Tibetan sheep, *Allium mongolicum* Regel powder, yeast cultures, rumen microbiota, 16S

## Abstract

The purpose of this experiment was to study the effect of *Allium mongolicum* Regel powder (AMR) and yeast cultures (YC) on rumen microbial diversity in Tibetan sheep in different Ecological niches. A total of 40 male Tibetan lambs with an initial weight of 18.56 ± 1.49 kg (6 months old) were selected and divided into four groups (10 sheep/pen; *n* = 10). In the Control Group, each animal was grazed for 8 h per day, in Group I, each animal was supplemented with 200 g of concentrate per day, in Group II, each animal was supplemented with 200 g of concentrate and 10 g of AMR per day, in Group III, each animal was supplemented with 200 g of concentrate and 20 g of YC per day. The experiment lasted 82 days and consisted of a 7-day per-feeding period and a 75-day formal period. The results indicated that at the phylum level, the abundance of Bacteroidota and Verrucomimicrobiota in L-Group II and L-Group III was increased, while the abundance of Proteobacteria was decreased in the LA (Liquid-Associated) groups. The proportion of F/B in S-Group II and S-Group III was increased compared to S-Group I and S-CON in the SA (Soild-Associated) group. At the genus level, the abundance of uncultured_rumen_bacterium and Eubacterium_ruminantium_group in L-Group II and L-Group III was increased. Furthermore, while the abundance of Rikenellaceae_RC9_gut_group was decreased in the LA, the abundance of Prevotella and Eubacterium_ruminantium_group was increased in the S-Group II and S-Group III compared to S-Group I and S-CON. The abundance of probable_genus_10 was the highest in S-Group II in the SA group. After the addition of YC and AMR, there was an increase in rumen microbial abundance, which was found to be beneficial for the stability of rumen flora and had a positive impact on rumen health.

## Introduction

In pursuit of higher breeding returns, antibiotics and antigenicity animal preparations are widely used in livestock and poultry production to improve feed conversion rate, anti-stress ability, and animal production efficiency (1). Resistance to bacterial pathogens is a result of the long-term use of antibiotic additives (2). Except for a small amount that can break down into non-toxic and harmless substances, 30–90% of antibiotics will enter the environment with excreta (3). Reaching a certain level of antibiotic residues can affect the structure of the microbial community, resulting in serious ecological imbalances that ultimately threaten human public health. Therefore, adding green additives such as plants, enzymes, and probiotics with regulatory activity to replace antibiotics is of great significance for improving animal productivity and rumen (4). At present, green additives that have been developed to replace antibiotics include microecological preparations, feed enzyme preparations, Chinese herbal medicine additives, and oligosaccharides (5, 6). *Allium mongolicum* Regel powder (AMR) has been widely studied for its safe and non-toxic side effects, improved feed palatability, and improved mutton flavor, which have led to increased productivity in ruminants (1). Research has found that providing garlic and its organic components to ruminants can help improve rumen fermentation and digestive capacity (7). Other research found that Allium plants can affect rumen fermentation (8). Previous research has shown that adding *Allium mongolicum* essential oil to the diet can effectively reduce the abundance of Bacteroidota flora, while simultaneously increasing the relative abundance of Firmicutes. This dietary modification also leads to an elevated Bacteroidota to Firmicutes ratio, which is known to promote improved energy utilization and facilitate animal growth and development (9, 10). Ethanol extract of *Allium mongolicum* was added to the diet and it was found that the abundance of Bacteroidota in the AME group was lower, while the abundance of Firmicutes, Proteobacteria, and Cyanobacterium was higher. At the genus level, it can reduce the abundance of bacterial genera related to the production of propionic acid, such as *Prevotella, Succinniclassicum, Selenomonas*, etc. In summary, *Allium mongolicum* and its extract have a certain regulatory effect on rumen fermentation and microbial flora. As a green additive for ruminants, *Allium mongolicum* and its extract have broad market prospects and huge potential value.

There has been a significant increase in concern over the effects of products containing active yeast and yeast cultures on rumen fermentation over the past few decades. Adding YC to feed can promote the digestion and absorption of nutrients by animals, reduce feed costs, and play an important role in maintaining animal health. It is increasingly being used to improve animal production performance and breeding efficiency (11). This study reveals that yeast cultures (YC) treated with high pressure (inactivated) do not have an effect on increasing the number of effective rumen bacteria (12, 13). However, YC that still have metabolic activity can successfully improve the activity of rumen microorganisms and increase the number of beneficial bacteria. Studies have shown that adding active dry yeast can improve the diversity and relative abundance of rumen microorganisms in fattening bulls (14). There are also studies showing that YC can increase microbial species abundance in the large intestine of calves, stimulate the colonization of fibrinolytic bacteria, and thus increase the production of butyrate (15).

Currently, the mechanism of the effects of YC and AMR on the production performance and rumen microbiota of ruminants is not clear, and there are differences in their application effects in production. Therefore, in-depth research on the effects of YC and AMR on the metabolism and physiological characteristics of gastrointestinal flora will contribute to a better understanding of the interaction between YC, *Allium mongolicum*, and gastrointestinal microorganisms in future research. In addition, it also helps us to develop green additives that can better improve the nutrition of anti-animals.

## Materials and methods

### Test materials

Yeast Culture (YC) was provided by an animal health limited company. The nutritional components of the product are as follows: Crude protein (CP) ≥ 12%, water ≤ 10%, Crude fiber (*CF*) ≤ 5%, Crude ash ≤ 3%, Ether extract (EE) ≥ 3%, *Saccharomyces cerevisiae* ≥ 2.0 × 108 CFU/g.

The preparation of Mongolian shallot Regel powder (AMR) involves drying and crushing fresh AMR purchased from the market. The preparation process is as follows. Place the fresh AMR flat on the clean and dry enamel plate and put it in the dry oven, set the temperature to 65°C, heat at a constant temperature for 12 h, treat with a 60–120 mesh crusher to form a powder, pass through an 80 mesh sieve, pack in plastic bags, and store at room temperature for future use. The nutritional content of AMR is shown in Table 1.

**Table 1:** The nutritional content of *Allium mongolicum* Regel powder.

Items	Content
DE/(MJ/kg)	12.71
ME/(MJ/kg)	10.47
Dry matter	93.40
Crude protein	22.34
Ether extract	4.68
Neutral detergent fiber	33.31
Acid detergent fiber	24.61
Calcium	1.40
Phosphorus	0.47

### Animals and experimental design

A total of 40 healthy male Tibetan sheep by Shangchuang Tibetan Sheep Husbandry Cooperative in Haidong, Qinghai Province were used as experimental animals. This study adopted a single-factor design for the experiments; forty male Tibetan sheep lambs with an initial weight of 18.56 ± 1.49 kg (6 months old) were selected and divided into four groups (10 sheep/pen; *n* = 10). Each animal in the control group was grazed for 8 h per day. The other groups were grazed for 8 h every day. In Experiment Group I, each animal was supplemented with 200 g of concentrate per day. In Experiment Group II, each animal was supplemented with 200 g of concentrate and 10 g of AMR per day (the amount of AMR added was based on the experimental results of Zhang Xiuyuan) (16). In Experiment Group III, each animal was supplemented with 200 g of concentrate and 20 g of YC per day (the amount of YC added was recommended in the instructions for use). The pre-trial period was 7 days, and the trial period was 75 days. Supplementary feed concentrate is produced by Lanzhou Zhengda Co., Ltd., and its composition and nutritional levels are shown in Table 2.

**Table 2:** Composition and nutrient levels of the supplemented concentrate after grazing (DM basis) %.

Ingredients	Content	Nutrient levels2)	Content
Soybean meal	5.00	DE/(MJ/kg)	12.04
Corn	50.00	ME/(MJ/kg)	9.87
Wheat bran	5.00	Dry matter	86.84
Cottonseed meal	3.00	Crude protein	14.89
Alfalfa hay	35.00	Ether extract	2.19
Limestone	0.50	Neutral detergent fiber	19.23
CaHPO4	0.60	Acid detergent fiber	14.26
NaCl	0.50	Calcium	1.08
Premix^1^)	0.40	Phosphorus	0.46
Total	100.00		

### Breeding management

Between October 2021 and January 2022, the experiment was conducted at the Shangchuang Tibetan Sheep Husbandry Cooperative in Haidong, Qinghai Province. Before the start of the experiment, the sheep shed was uniformly cleaned and disinfected. After deworming the sheep, the vaccine was injected according to the normal immune procedure, and the ear labels of the test sheep were registered. The sheep were divided into four columns according to their groups, with 10 males in each column for feeding. Daily environmental management of the sheep shed was strengthened. During the test, in addition to carrying out regular feeding, it was important to carefully observe the test sheep’s daily feeding and water intake to ensure normalcy. Additionally, it is crucial to monitor rumination patterns, fecal consistency, and the mental well-being of the test subjects. Keep the feeding environment and conditions of each group consistent during the test period. To ensure that the test sheep could receive a certain amount of AMR and YC, the AMR or YC was mixed with a small amount of concentrate before the sheep could freely feed. To ensure free drinking during this time, it is recommended to add extra supplements after testing the sheep.

### Sample collection and index determination

Fresh rumen contents from slaughtered Tibetan sheep were collected following the sampling requirements of microbial testing after the experiment. We mainly collected samples from the rumen and transferred them to sterile frozen storage tubes. We immediately placed them in liquid nitrogen and transferred them to −80°C ultra-low temperature refrigerators for analysis of rumen microbiota.

#### Sample pretreatment

To pretreat the rumen liquid-associated (LA) microbiota, the filtered rumen fluid has been centrifuged at 10,000 rpm for 20 min at room temperature. After discarding the supernatant, the precipitate was collected. A small amount of physiological saline was used to suspend the precipitate, which served as a rumen liquid phase microorganism. Finally, the suspension was stored at −80°C.

To pretreat the rumen liquid-associated (LA) microbiota, 50 g (based on actual weight) of solid sample was transferred to a centrifuge tube and 150 mL (based on actual weight) of sterilized physiological saline was added to resuspend plant particles. The mixture was shaken and mixed well for 30 s and then centrifuged at 350 rpm for 15 min at room temperature to precipitate plant particles. The supernatant was carefully removed, transferred to a new tube (marked as S), and then placed at 4°C or on ice. The recovered sediment particles were suspended again in 25 mL (depending on the actual weight) of anaerobic paint thinner containing 0.15% (v /v) Tween-80. The mixture was shaken and mixed for 30 s, then put on ice for 2.5 h to elute closely attached bacteria. The eluted mixture system was centrifuged at 350 rpm for 15 min at room temperature to remove plant particles. The filtrate was mixed with the supernatant in the S bottle and centrifuged at 4°C, at 10000 rpm for 20 min. The precipitated portion was the recovered solid microbial cells; the sediment was resuspended in the smallest volume of sterilized physiological saline, which was the rumen solid-phase microorganism, and stored for analysis at −80°C.

In order to distinguish the groups after different treatments, the LA test groups were named separately as L-CON, L-Group I, L-Group II, and L-Group III, and the SA test groups were named separately as S-CON, S-Group I, S-Group II, and S-Group III.

#### Measurement indicators and methods

The TGuide S96 magnetic bead method soil /fecal genome DNA extraction kit (Tiangen Biotechnology (Beijing, China) Co., Ltd.) was used to extract total microbial DNA from Tibetan sheep rumen samples. After extraction, the microplate reader (manufacturer: Gene Company Limited, model synergy HTX) was used to detect the concentration of nucleic acid, according to the detection and amplification, and 1.8% agarose was used to detect the integrity of the PCR products after amplification. Following extraction, the concentration of nucleic acid was determined using a microplate reader (manufacturer: Gene Company Limited, model: synergy HTX). The integrity of the PCR products obtained from the amplification was assessed using 1.8% agarose gel electrophoresis. Specific primers containing barcodes for the V1–V9 variable regions of 16S rRNA genes of bacteria that meet both total and quality standards were designed. Veriti96well9902 (Applied Biosystems, Foster City, CA, United States) PCR instruments and a 16S full-length reaction system (30 μ L system) for PCR amplification were used (Table 3). The specific primer sequence was 27F_(16S-F) (5’-AGRGTTTGATYNTGGCTCAG-3′) and1492R_(16S-R) (5’-TASGGHTACCTTGTTASGACTT-3′). Illumina MiSeq (Illumina Inc. San Diego, CA, United States) sequencing and data analysis were completed by Biotech Co., Ltd. (Beijing, China). After extracting the total DNA samples, specific primers with barcodes were synthesized based on the full-length primer sequences for PCR amplification. The resulting products were purified, quantified, and normalized to form sequencing libraries (SMRT Bell). The constructed libraries underwent quality inspection, and those that passed the quality check were sequenced using PacBio Sequel II. The data generated by PacBio Sequel II was in BAM format, and the CCS files were exported using the SMRT Link analysis software. The data was then identified based on the barcode sequences to differentiate the samples and transformed into FASTQ format. The sequenced sequences were subjected to OTU (Operational Taxonomic Units) clustering and bioinformatics statistical analyses using software such as Mothur (version v.1.30.1) and Usearch (version 7.0), with a similarity of 97%.

**Table 3:** 16S full-length reaction synthesis (30 μL synthesis).

	Dosage (μl)
Genomic DNA	1.5
NFW	10.5
KOD ONE MM	15
Barcode primer pair	3
Overall system	30

### Statistical analysis

Taxonomy annotation of the OTUs/ASVs was performed based on the Naive Bayes classifier in QIIME2 (17) using the SILVA database (release 138.1) with a confidence threshold of 70%. The Alpha diversity was calculated and displayed using QIIME2 and R software. Beta diversity was determined to evaluate the degree of similarity of microbial communities from different samples using QIIME2. Principal coordinate analysis (PCoA), heatmaps, UPGMA, and nonmetric multidimensional scaling (NMDS) were used to analyze the beta diversity. Furthermore, we employed Linear Discriminant Analysis (LDA) effect size (LEfSe) (18) to test the significant taxonomic differences between groups. A logarithmic LDA score of 4.0 was set as the threshold for discriminative features. In addition, at the phylum, class, order, family, genus, and specifications levels, the composition of each sample community was statistically analyzed. Meanwhile, the charting was performed using the online platform.

## Results

In the LA and SA groups, a total of 370,848 CCS sequences were obtained after 48 samples were sequenced and identified by barcode. Each sample produced at least 6,760 CCS sequences, with an average of 7,726 CCS sequences (Supplementary Table S1). The effective tags of all samples were clustered with 97% identity, and a total of 1,488 OTUs were obtained. The representative sequences of OTUs were selected for species annotation, and a total of 19 phyla, 32 classes, 55 orders, 108 families, 209 genera, and 341 species were annotated in all samples (Supplementary Table S2).

### Effects of AMR and YC on Alpha diversity of liquid-associated and solid-associated microbiota in rumen

**Figure 1:**
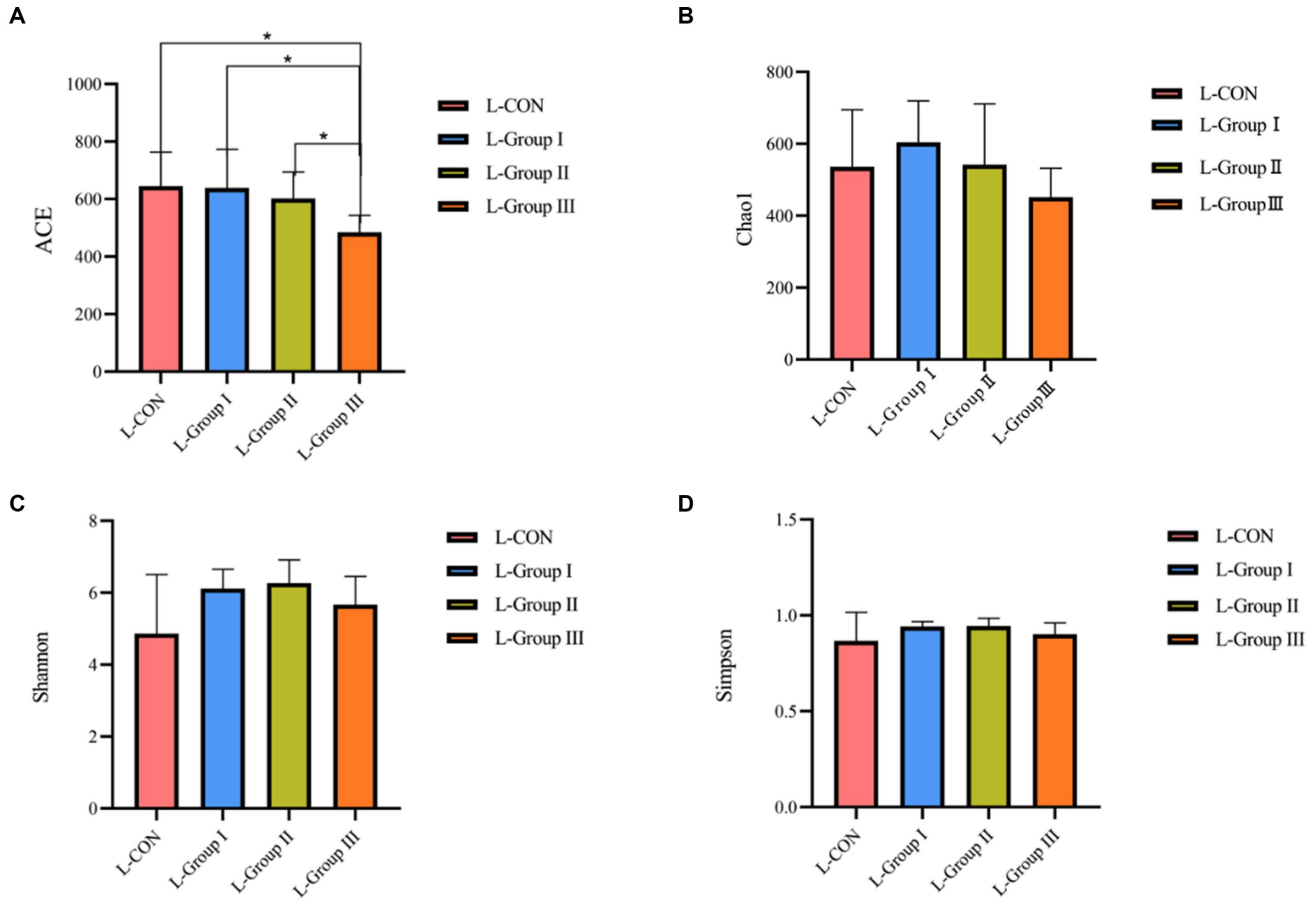
Effects of AMR and YC on Alpha diversity index of rumen LA microorganisms. ACE **(A)**, Chao1 **(B)**, Shannon **(C)**, and Simpson **(D)**, respectively.

**Figure 2:**
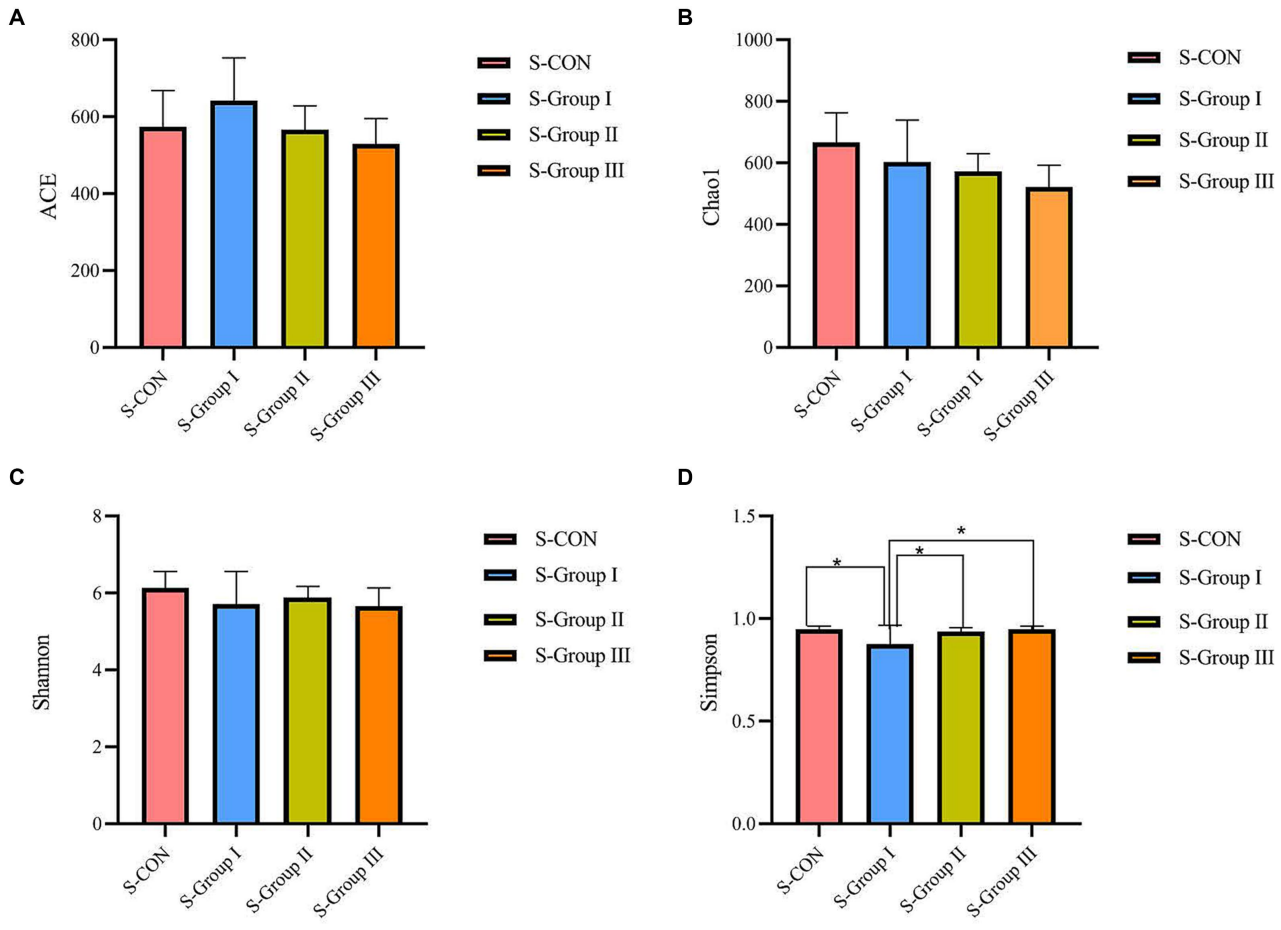
Effects of AMR and YC on Alpha diversity index of rumen SA microorganisms. ACE **(A)**, Chao1 **(B)**, Shannon **(C)**, and Simpson **(D)**, respectively.

According to the Alpha diversity analysis of OTU based on the 16 s results of rumen microorganisms, In the rumen LA microorganisms, significant differences (*p* < 0.05) were observed in the ACE and Chao1 indexes among the four experimental groups. The ACE index and Chao1 index of the L-CON, L-Group I, and L-Group II experimental groups were significantly higher than those of L-Group III. The Simpson index and Shannon index did not show significant differences between the groups (Figure 1). As shown in Figure 2, according to the OTU analysis of 16 s rumen microbial detection results, the Alpha diversity analysis of rumen SA microorganisms revealed no significant differences in the ACE index, Chao1 index, and Shannon index among the four experimental groups. However, there were significant differences observed in the Simpson index between the groups. The Simpson index of the S-CON, S-Group II, and S-Group III was significantly higher than that of S-Group I.

**Figure 3:**
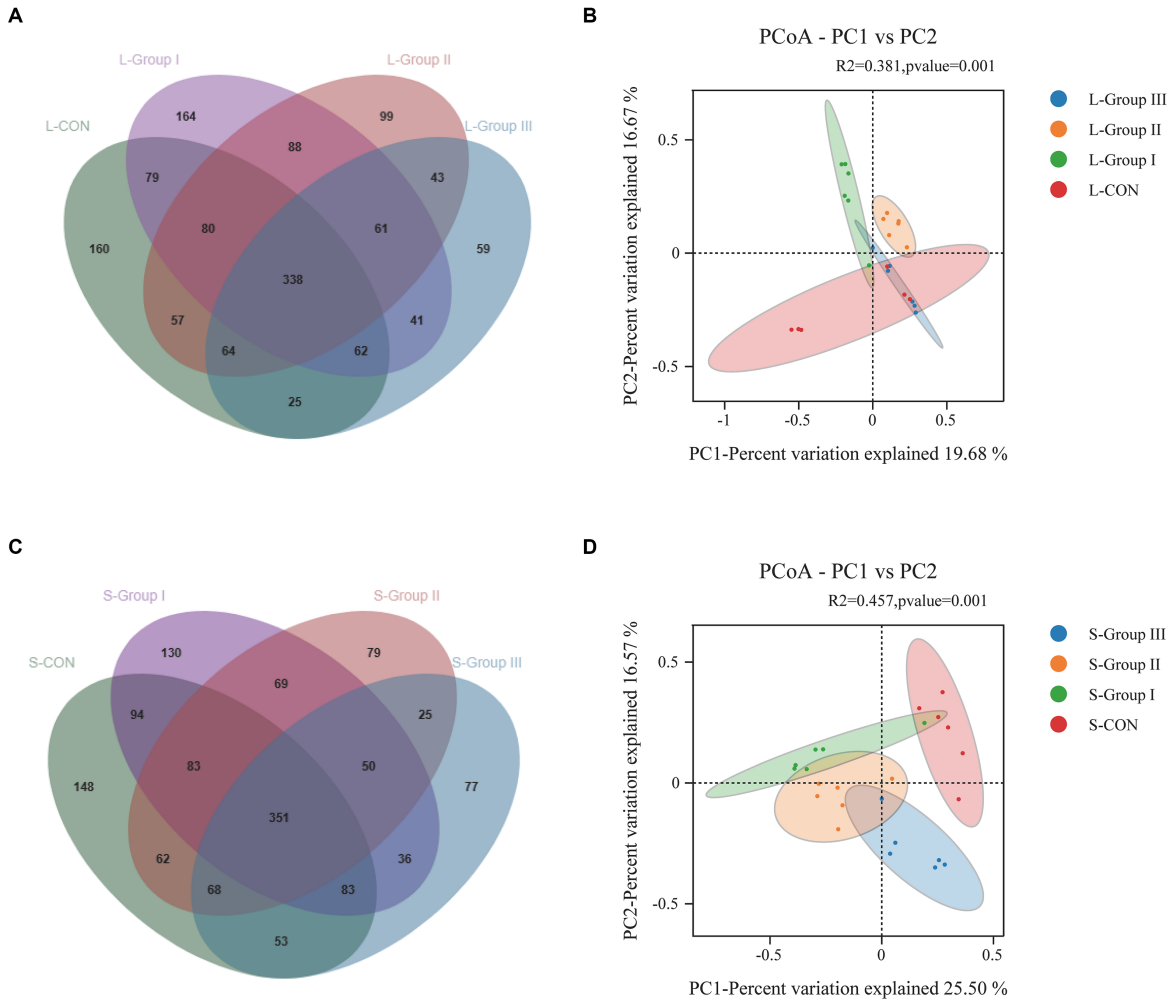
Rumen LA microorganisms Venn **(A)**, PCoA **(B)**, and rumen SA microorganisms Venn **(C)**, PCoA **(D)**. Different colors of Venn and PCoA represent different sample groups.

As shown in Figure 3A, there were 338 OTUs in the rumen LA flora of the four experimental groups, of which 160 were L-CON specific OTUs, 164 were L-Group I specific OTUs, 99 were L-Group II specific OUTs, and 59 were L-Group III specific OTUs. The principal coordinate analysis of species abundance data at the OTU level shows that the coefficients of PCoA plot samples PC1 and PC2 were, respectively, 19.68 and 15.75%. It was observed that there was a higher degree of individual aggregation between L-CON and L-Group III, suggesting a higher similarity in microbial community structure in this group compared to other sample types. Additionally, the samples within this group exhibited close proximity to each other. The microbial community structure of L-Group I and L-Group II was relatively independent of L-CON; in addition, a significance test (R2 = 0.381, *p* = 0.001) was conducted in conjunction with PERMANOVA, indicating differences in the structure of rumen LA microbial communities (Figure 3B). According to Figure 3C, there were 351 OTUs in the rumen SA flora of the four experimental groups, of which 148 were S-CON specific OTUs, 130 were S-Group I specific OTUs, 79 were S-Group II specific OTUs, and 77 were S-Group III specific OTUs. The principal coordinate analysis was based on the species abundance data at the OTU level. The PCoA plot samples exhibited coefficients of 25.50 and 16.57% for PC1 and PC2, respectively. The bacterial communities in S-CON, S-Group I, S-Group II, and S-Group III appear to be relatively independent. A significance test (R2 = 0.457, *p* = 0.001) was conducted in conjunction with PERMANOVA, indicating differences in the structure of rumen liquid phase microbial communities (Figure 3D).

### Effects of AMR and YC on beta diversity of liquid-associated and solid-associated microbiota in rumen

**Figure 4:**
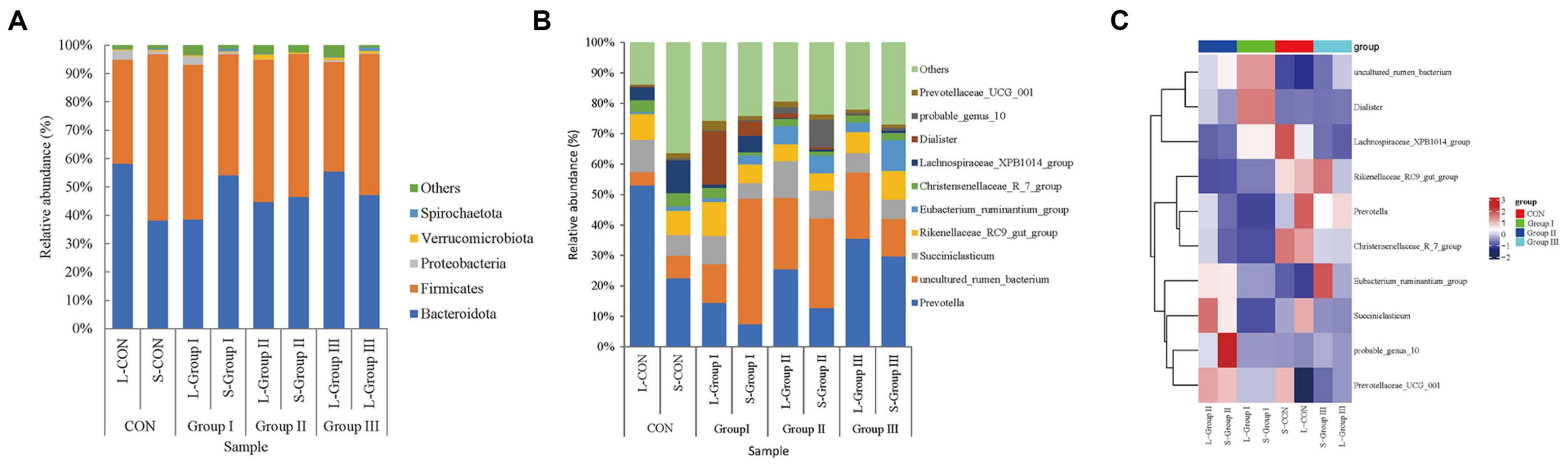
Bar chart of relative abundance at the phylum level **(A)**, genus level **(B)**, and Rumen LA and SA microbial heatmap **(C)**. Different colors in the bar chart represent species abundance.

The main differences in the phylum level of rumen bacteria between the four groups are shown in Figure 4A. Bacteroidota and Firmicutes are the two dominant bacteria in Phylum with microbial abundance higher than 1% at the level of rumen LA microbiota. The relative abundance of Bacteroidota and Firmicutes in the L-CON, L-Group I, L-Group II, and L-Group III were 58.23, 38.61, 44.71, and 55.49% and 36.75, 54.57, 50.22, and 38.62%, respectively, with significant differences between the groups (*p* < 0.05). The abundance of Verrucomimicrobiota significantly increased while Proteobacteria decreased in L-Group II. In SA rumen microorganisms, the two dominant strains are Bacteroidota and Firmicutes. The relative abundance ratio of Firmicutes in the four experimental groups was as follows: S-CON>S-Group II > S-Group III > S-Group I, with values of 58.66, 50.53, 49.75, and 42.72%, respectively. On the other hand, the relative abundance ratio of Bacteroidota in the four experimental groups was as follows: S-Group I > S-Group III > S-Group II > S-CON, with values of 54.14, 47.23, 46.50, and 38.23%, respectively. The relative abundance of the two dominant Phylum in this experiment showed a significant negative correlation (Figure 4A).

At the genus level, in LA rumen microorganisms, the abundance of *Prevotella* and *Lachnospiraceae_XPB1014_group* in L-CON was significantly higher than in L-Group I, L-Group II, and L-Group III (*p* < 0.05). Additionally, the abundance of *probable_genus_10* in L-Group I was significantly higher than in L-CON, L-Group II, and L-Group III (*p* < 0.05). Moreover, the abundance of *Prevotellaceae_UCG_001* in L-Group II was significantly higher than in L-CON, L-Group I, and L-Group III (*p* < 0.05). Furthermore, the abundance of *uncultured_rumen_bacterium* in L-Group II and L-Group III was significantly higher than in L-CON and L-Group I (*p* < 0.05) (Figure 4B). In SA rumen microorganisms, the abundance of *Christensenellaceae_R_7_group* and *Lachnospiraceae_XPB1014_group* in S-CON was significantly higher than in S-Group I, S-Group II, and S-Group III (*p* < 0.05). The abundance of *Dialisrer* and *uncultured_rumen_bacterium* in S-Group I was significantly higher than in S-CON, S-Group II, and S-Group III (*p* < 0.05). Furthermore, the abundance of *probable_genus_10* in S-Group II was significantly higher than in S-CON, S-Group I, and S-Group III (*p* < 0.05). Lastly, the abundance of *Eubacterium_ruminantium_group* in S-Group III was significantly higher than in S-CON, S-Group I, and S-Group II (*p* < 0.05; Figure 4B).

At the genus level, the abundance of *Prevotella* was significantly higher in L-CON than in S-CON; the abundance of *uncultured_rumen_bacterium*, *Lachnospiraceae_XPB1014_group,* and *Succiniclasticum* was higher in S-CON than in L-CON; the abundance of *Prevotella, Rikenellaceae_RC9_gut_group,* and *Dialister* was higher in L-Group I than in S-Group I; the abundance of *uncultured_rumen_bacterium* was higher in S-Group I than in L-Group I; the abundance of *Prevotella* and *Succiniclasticum* was higher in L-Group II than in S-Group II, but the abundance of *probable_genus_10* was higher in S-Group II than in L-Group II; the abundance of *Prevotella* and *uncultured_rumen_bacterium* was higher in L-Group III than in S-Group III; and the abundance of *Rikenellaceae_RC9_gut_group* and *Eubacterium_ruminantium_group* was higher in S-Group III than in L-Group III. Interestingly*, Prevotella* was higher in the LA groups than in the SA groups (Figure 4B). The abundance of *probable_genus_ 10* was highest in S-Group II and the abundance of *Prevotelaceae_ UCG_ 001* was lowest in L-CON in the heat map (Figure 4C).

**Figure 5:**
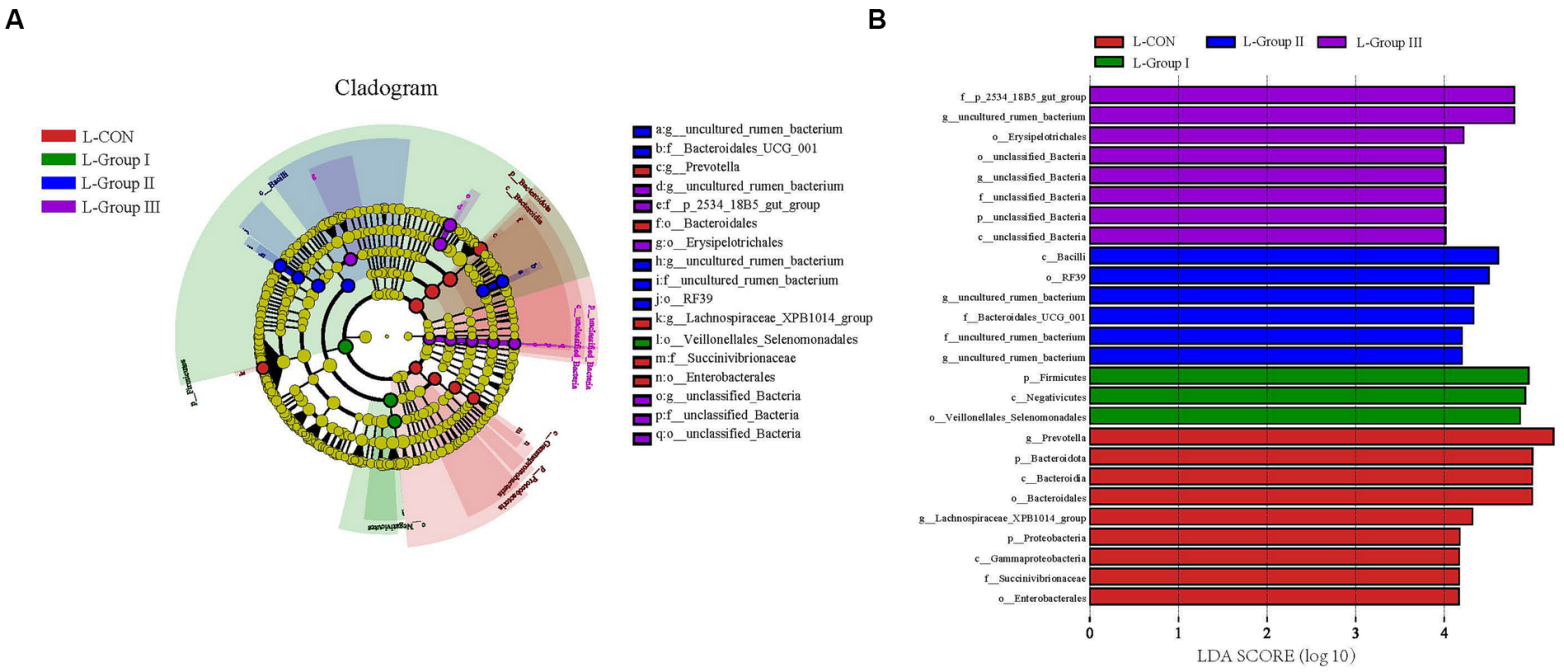
Microbial communities of different groups. The LEfSe analysis cladogram of LA microorganisms **(A)**. Linear discriminant analysis (LDA) was used to estimate the effect of each component (species) abundance on the difference **(B)**. Species with an LDA Score greater than a set value (set to 4.0 by default) are shown, with the length of the bars representing the effect size of the differing species (i.e., as an LDA logarithmic score), and the different colors of the bar graph indicate the sample group corresponding to the taxon with higher abundance.

The LefSe Cladogram and LDA (Score > 4.0) in Figure 5 reveal significant differences in certain species between the L-CON and experimental groups. The use of concentrates and additives can cause changes in the microbiota in the rumen LA microbiota.

**Figure 6:**
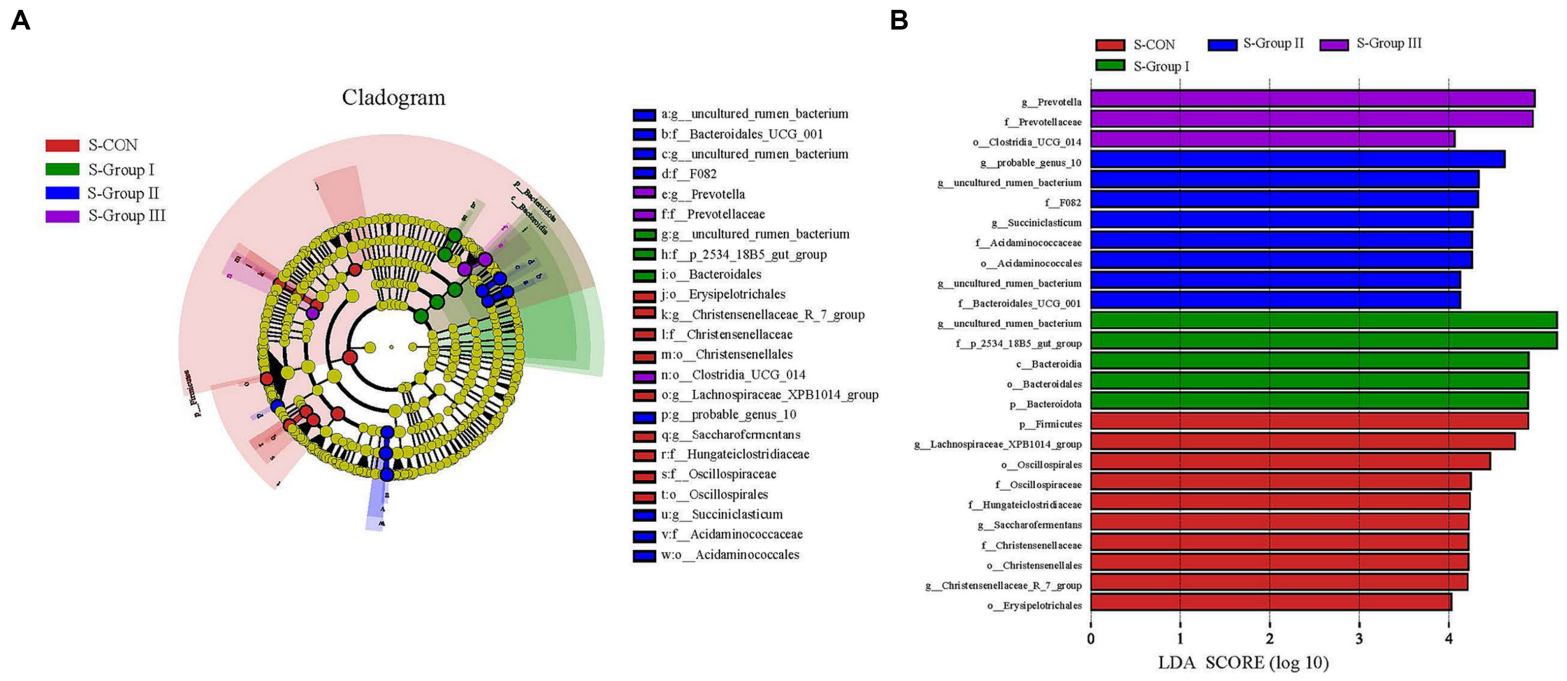
Microbial communities of different groups. The LEfSe analysis cladogram of SA microorganisms **(A)**. Linear discriminant analysis (LDA) was used to estimate the effect of each component (species) abundance on the difference **(B)**. Species with an LDA Score greater than a set value (set to 4.0 by default) are shown, with the length of the bars representing the effect size of the differing species (i.e., as an LDA logarithmic score), and the different colors of the bar graph indicate the sample group corresponding to the taxon with higher abundance.

Setting the LDA threshold to 4 and comparing the differences between the four groups of microbial communities using LEfSe (Figure 6) shows that there are differences in the significantly enriched microbial genera between different experimental groups of SA rumen microbiota.

## Discussion

Generally, the common method for studying rumen microorganisms is to collect rumen fluid. However, different rumen sites, including rumen epithelium, rumen juice, and solid rumen chyme, may have different microorganisms (19, 20). A previous study based on 16 s Ribosome RNA (rRNA) gene sequencing showed that there was no difference in the classification composition between the solid adhesion environment and the liquid environment in the rumen, which was mainly distinguished by the relative abundance of species (21).

In this study, the ACE index and the Chao1 index in the Alpha diversity analysis were significantly different among the LA groups. The ACE Index of L-CON and L-Group I was significantly higher than that of the experimental L-Group III group, and the Chao1 Index of L-CON, L-Group I, and L-Group II was significantly higher than that of L-Group III. The Simpson Index and Shannon Index showed no significant difference, indicating that the addition of AMR and YC had a significant impact on the abundance of rumen LA microorganisms. The ACE and Chao1 indexes in the SA groups were not significant in the test group, indicating that the addition of AMR and YC had little effect on the richness of SA microorganisms in the rumen of Tibetan sheep. In comparison, the Simpson index of S-CON, S-Group II, and S-Group III was 1.13, 1.12, and 1.08 times that of S-Group I, respectively. The Simpson index of S-Group I is the smallest, indicating that the effectiveness of the two additives is to some extent consistent with the grazing feeding mode on microbial diversity in the rumen. At the same time, it was found that L-Group I, which originally had the highest Simpson index among rumen LA microorganisms, showed the lowest Alpha diversity analysis among SA microorganisms. This phenomenon was also observed in the SA microorganisms of other test groups when compared with the LA microorganisms. This observation suggests that the rumen is an important factor in maintaining gut microbiota stability. To adapt to the changes in the environment and feed, the microbial abundance in different Ecological niches has not changed. However, the diversity of its flora will always remain in a certain dynamic equilibrium to ensure the normal operation of the rumen of animals.

In this experiment, PCoA analysis of rumen microbiota in the LA groups showed that the coordinates of L-Group II and L-Group III microbiota were closer, while L-CON and L-Group I showed more dispersion. The results demonstrate that the addition of AMR and YC to the diet increased the similarity of microbiota among Tibetan sheep samples. This suggests that AMR and YC are conducive to the stability of rumen microbial communities in Tibetan sheep. Bacteroidota is directly related to the degradation of non-fibrous nutrients, and its main function is to improve the utilization rate of carbohydrates. It can also improve immune function and inhibit inflammatory reaction occurrence (22), Firmicutes mainly degrade fibrous substances and promote the fermentation function of the rumen on feed (23). Bacteroidota and Firmicutes as dominant microbiota widely present in ruminants (24, 25). The two dominant phyla in this experiment are consistent with the results of this study. The abundance of Bacteroidota in L-Group II and L-Group III, with the addition of AMR and YC was higher than in L-Group I in the LA group. This indicates that the addition of AMR and YC contributes to the decomposition of non-fibrous substances in the rumen of Tibetan sheep fed in captivity. This result is consistent with the study on the effect of adding scallions to the diet on the rumen microbiota of cattle (26). The study on adding YC to sheep feed showed that it can increase the relative abundance of Bacteroidota without having a significant impact on other phylum. This is consistent with the results of this experiment. The experimental group with added YC showed an increase in the abundance of Bacteroidota in the LA groups (27). The microbial abundance of Bacteroidota in L-CON was significantly higher than that of other test groups, while the abundance of Firmicutes was significantly lower. This suggests that grazing can improve the growth and proliferation of non-fiber-degrading bacteria in the rumen. This result is similar to other studies (28). With the addition of concentrate, AMR and YC resulted in a gradual increase in the abundance of Bacteroidota in L-Group I, L-Group II, L-Group III, S-Group I, S-Group II, and S-Group III. It may be that after feeding the concentrate to Tibetan sheep, the non-fibrous nutrients such as soybean meal, corn, and wheat bran in the concentrate increased the number of Bacteroidota. At the same time, the abundance of Spirochaete in the rumen of L-Group II and L-Group III was higher compared to L-Group I, and the abundance of Proteobacteria flora was reduced. Numerous studies have revealed that the Proteobacteria in the intestine can reflect the imbalance or unstable structure of the microecology, and the metabolic disorder of the microecology is often accompanied by the increase of the Proteobacteria. The use of two green additives, AMR and YC, improves the changes in rumen microbiota at the phylum level while contributing to maintaining the homeostasis of rumen microorganisms. An increasing number of studies have shown that different nutrients affect the digestive capacity of rumen microorganisms (29). Bacteroidota and Firmicutes are the dominant bacteria in the rumen, and their ratios (F/B ratio for short) may better reflect the physiological state of rumen flora (30). Compared to other groups, the addition of AMR did not improve the abundance of Spirochaete and Fibrobacterota (31). Spirochaete in S-Group II had more significant effects on the SA microorganisms than the other test groups, which might be based on different Ecological niches in the rumen in this experiment. Adding AMR to the diet can provide growth nutrients for these two bacteria. Verrucomimicrobia is a vital factor affecting mammalian immune function, and the decrease in its abundance is concerned with the decline in the immune function of the body (32). This indicates that the addition of YC can not only maintain the stable structure of the microbiota but also improve the immune ability of Tibetan sheep.

At the genus level, *Prevotella*, which belongs to *Bacteroidota*, is a widely existing bacterium in the rumen. Its main role is to participate in semi-fiber degradation and other components in the diet and various metabolic activities in the rumen. *Prevotella* produces a large number of complex enzymes, facilitating starch decomposition and some cell wall polysaccharides (33, 34). In the LA groups, the abundance of *Prevotella* in the L-CON was significantly higher than in other test groups. The abundance of *Prevotella* in L-Group I, L-Group II, and L-Group III showed a gradually increasing trend, which was similar to the Species distribution trend of *Bacteroidota* at the phylum level. Studies have also found that compared to calves fed with concentrate, calves fed with forage had a higher abundance of *Prevotella* in the rumen (35). This suggests that traditional grazing feeding models are more conducive to the growth and proliferation of LA microorganisms in the rumen compared to those fed with concentrate after grazing. The study found that differences in crude protein levels, neutral detergent fibers, and acidic detergent fibers in the diet can all cause differences in the species and abundance of *Prevotella* in the rumen of ruminants (36). Compared with L-Group II and L-Group III, the content of *Prevotella* in L-Group I increased, possibly due to the nutritional components of additives causing changes in the abundance of LA microbiota in the rumen. In the SA groups, the addition of YC resulted in an increase in the abundance of *Prevotella* and *Eubacterium ruminantium*, both of which belong to Firmicutes’ S-Group III. This indicates that YC can improve the degradation of rumen cellulose in the SA groups (37). However, the specific functions of YC in this regard require further investigation. *Prevotelaceae_ UCG_ 001* as the main bacterial branch of *Bacteroidota*, is mainly responsible for the decomposition of Hemicellulose, carbohydrate, and protein. It exhibits the highest abundance in L-Group II, indicating that the addition of AMR can improve the efficiency of Tibetan sheep in the decomposition of hemicellulose to a certain extent. In addition, the inclusion of AMR increased the abundance of *problem_genus_10* in the SA system, indicating that the use of additives in feed can effectively improve the abundance of SA system microorganisms in the rumen. *Christenselelaceae_ R_ 7_ Group* belonging to Firmicutes, is positively related to the generation and transport of volatile acids in the rumen. It also promotes the degradation of fiber materials and improves the utilization rate of forage. Adding AMR to the diet of Tibetan sheep resulted in an improvement in the levels of *Prevotella* and *uncultured_rumen_bacterium* in the rumen microbiota. However, it also caused a decrease in the abundance of *Rikenellaceae_RC9_gut_group*. The decrease could be attributed to the high content of crude protein and fat in scallions, which enhances the degradation of hemifiber and other dietary components, as well as various metabolic activities in the rumen. These processes lead to the production of complex enzymes, starch, and some cell wall polysaccharides (33, 34), ultimately resulting in an increase in the abundance of LA microbial *uncultured_rumen_bacterium.*

Furthermore, the combination of LEfSe analysis and the comparison between groups revealed notable changes in the structure and abundance of the core rumen flora in Tibetan sheep when concentrate, AMR, and YC were added to the LA groups. These findings have important implications for regulating the rumen flora environment in production, as they highlight the potential benefits of using effective additives and concentrate in this experiment. In addition, the analysis of LEfSe and LDA column plots in the SA system showed that the addition of AMR and YC had an impact on the core microbial community in the rumen. Specific bacterial genera showed noteworthy differences compared to other groups. These findings suggest that supplementary feeding alone may not fully account for these results, as factors such as animal species and levels of added concentrate may contribute to variations in the core microbiota and its similarity.

## Conclusion

In both the LA and SA systems, the addition of AMR to the diet resulted in an improvement in the abundance of beneficial flora, such as Succiniclasticum, which aids in cellulose decomposition. This addition also increased the abundance of immune regulation and inflammatory suppression bacteria, such as *Verrucomicrobiota* and *Eubacterium_ruminantium_group*, while reducing the presence of unfavorable microbiota, including *Proteobacteria* and *Rikenellaceae_RC9_gut_group*. These changes ultimately promoted the stability of rumen microbiota. Similarly, the addition of YC increased the abundance of non-fiber degrading bacteria, such as Bacteroidota and Spirochaetota, while reducing the presence of pathogenic bacteria, such as *Proteobacteria* and *Rikenellaceae_RC9_gut_group*. This supplementation not only enhanced the immunity of Tibetan sheep but also contributed to the stability of the rumen microbiota structure. Based on these results, we concluded that adding AMR and YC to the diet had a beneficial effect on rumen health. This provides a new perspective on the stability of feed additives on rumen microbial flora and provides new insights into the role of AMR and YC in diets.

## Data availability statement

The original contributions presented in the study are publicly available. This data can be found at: https://www.ncbi.nlm.nih.gov/; PRJNA1018211.

## Ethics statement

The animal study was approved by Animal Care Committee of Gansu Agricultural University (GSAU-AEW-2020-0057). The study was conducted in accordance with the local legislation and institutional requirements.

## Author contributions

CW: Conceptualization, Data curation, Formal analysis, Investigation, Software, Writing – original draft, Writing – review & editing. JF: Formal analysis, Investigation, Methodology, Writing – review & editing. KM: Writing – review & editing. HW: Writing – review & editing. DL: Writing – review & editing. TL: Writing – review & editing. YM: Funding acquisition, Supervision, Writing – review & editing.

## References

[1] Kim ET Guan LL Lee SJ Lee SM Lee SS Lee ID. Effects of flavonoid-rich Plant extracts on in vitro ruminal Methanogenesis, microbial populations and fermentation characteristics. Asian Australas J Anim Sci. (2015) 28:530–7. doi: 10.5713/ajas.14.0692, PMID: 25656200 PMC4341102

[2] Hernandez A Kholif AE Lugo-Coyote R Elghandour MMY Cipriano M Rodríguez GB. The effect of garlic oil, xylanase enzyme and yeast on biomethane and carbon dioxide production from 60-d old Holstein dairy calves fed a high concentrate diet. J Clean Prod. (2017) 142:2384–92. (Article). doi: 10.1016/j.jclepro.2016.11.036

[3] Ayukekbong JA Ntemgwa M Atabe AN. The threat of antimicrobial resistance in developing countries: causes and control strategies. Asian Pac J Trop Med. (2017) 6:47. doi: 10.1186/s13756-017-0208-x, PMID: 28515903 PMC5433038

[4] Cedillo J Kholif AE Salem AZM Elghandour MMY Vázquez JF Alonso MU. Oral administration of sauce llorOn extract to growing lambs to control gastrointestinal nematodes and Moniezia spp. Asian Pac J Trop Med. (2015) 8:520–5. doi: 10.1016/j.apjtm.2015.06.011 26276281

[5] Korotkova OG Rozhkova AM Kislitsin VY Sinitsyna OA Denisenko YA Marochkina MA. New feed enzyme preparations for the destruction of nonstarch polysaccharides and Phytates. Mosc Univ Chem Bull. (2023) 78:63–8. doi: 10.3103/s0027131423020037

[6] Shi XH Jia YY Zhang ZF Wu W Wu Z Chi M. The effects of Chinese herbal feed additives on physiological health and detoxification ability in the red claw crayfish, Cherax quadricarinatus, and evaluation of their safety. Aquaculture. (2023) 569:739394. doi: 10.1016/j.aquaculture.2023.739394

[7] Anassori E Dalir-Naghadeh B Pirmohammadi R Taghizadeh A Asri-Rezaei S Maham M. Garlic: a potential alternative for monensin as a rumen modifier. Livest Sci. (2011) 142:276–87. doi: 10.1016/j.livsci.2011.08.003

[8] Wanapat M Khejornsart P Pakdee P Wanapat S. Effect of supplementation of garlic powder on rumen ecology and digestibility of nutrients in ruminants. J Sci Food Agric. (2008) 88:2231–7. doi: 10.1002/jsfa.3333

[9] Yaxing Z Erdene K Zhibi B Changjin A Chen B. Effects of Allium mongolicum regel essential oil supplementation on growth performance, nutrient digestibility, rumen fermentation, and bacterial communities in sheep. Front Vet Sci. (2022) 9:9. doi: 10.3389/fvets.2022.926721, PMID: 36387406 PMC9659749

[10] Zhao Y Zhang Y Khas E Ao C Bai C. Effects of Allium mongolicum regel ethanol extract on three flavor-related rumen branched-chain fatty acids, rumen fermentation and rumen bacteria in lambs. Front Microbiol. (2022) 13:13. doi: 10.3389/fmicb.2022.978057, PMID: 36187944 PMC9520700

[11] Chademana IZ. The effect of dietary inclusion of yeast culture on digestion in sheep. Anim Sci. (1989) 50:483–9. doi: 10.1017/S0003356100004967

[12] Dawson KA Newman KE Boling JA. Effects of microbial supplements containing yeast and lactobacilli on roughage-fed ruminal microbial activities. J Anim Sci. (1990) 68:3392–8. doi: 10.2527/1990.68103392x, PMID: 2123850

[13] El Hassan SM Newbold CJ Wallace RJ. The effect of yeast culture on rumen fermentation: growth of the yeast in the rumen and the requirement for viable yeast cells. Trop Anim Health Prod. (1972) 1993:175–5. doi: 10.1017/s0308229600024971

[14] Gao K Geng C. Alterations in the rumen bacterial communities and metabolites of finishing bulls fed high-concentrate diets supplemented with active dry yeast and yeast culture. Front Microbiol. (2022) 13:13. doi: 10.3389/fmicb.2022.908244, PMID: 36605509 PMC9810264

[15] Alugongo GM Xiao JX Chung YH Dong SZ Li SL Yoon I. Effects of *Saccharomyces cerevisiae* fermentation products on dairy calves: performance and health. J Dairy Sci. (2017) 100:1189–99. doi: 10.3168/jds.2016-11399, PMID: 28012624

[16] Zhang XY Li SY Wang CF Ding H Ao C. Effects of sand onion and its extracts on growth performance and fat metabolism-related indexes of little-tailed cold sheep. Chin J Anim Nutr. (2019) 31:334–41. doi: 10.3969/j.issn.1006-267x.2019.01.040

[17] Bolyen E Rideout JR Dillon MR Bokulich NA Abnet CC al-Ghalith GA. Reproducible, interactive, scalable and extensible microbiome data science using QIIME 2. Nat Biotechnol. (2019) 37:852–7. doi: 10.1038/s41587-019-0209-9, PMID: 31341288 PMC7015180

[18] Segata N Izard J Waldron L Gevers D Miropolsky L Garrett WS. Metagenomic biomarker discovery and explanation. Genome Biol. (2011) 12:R60–18. doi: 10.1186/gb-2011-12-6-r60, PMID: 21702898 PMC3218848

[19] Kong YH Teather R Forster R. Composition, spatial distribution, and diversity of the bacterial communities in the rumen of cows fed different forages. FEMS Microbiol Ecol. (2010) 74:612–22. doi: 10.1111/j.1574-6941.2010.00977.x, PMID: 21044097

[20] Mann E Wetzels SU Wagner M Zebeli Q Schmitz-Esser S. Metatranscriptome sequencing reveals insights into the gene expression and functional potential of Rumen Wall Bacteria. Front Microbiol. (2018) 9:43. doi: 10.3389/fmicb.2018.00043, PMID: 29410661 PMC5787071

[21] De Mulder T Goossens K Peiren N Vandaele L Haegeman A De Tender C. Exploring the methanogen and bacterial communities of rumen environments: solid adherent, fluid and epimural. FEMS Microbiol Ecol. (2017) 93:fiw251. doi: 10.1093/femsec/fiw251, PMID: 28011597

[22] Xu J. Degradation characteristics of NDF from different forage sources in rumen and its effect on bacterial community structure. PHD theise. Yangzhou, China: Yangzhou University (2014).

[23] Evans NJ Brown JM Murray RD Getty B Birtles RJ Hart CA. Characterization of novel bovine gastrointestinal tract Treponema isolates and comparison with bovine digital dermatitis Treponemes. Appl Environ Microbiol. (2011) 77:138–47. doi: 10.1128/AEM.00993-10, PMID: 21057019 PMC3019712

[24] Ley RE Lozupone CA Hamady M Knight R Gordon JI. Worlds within worlds: evolution of the vertebrate gut microbiota. Nat Rev Microbiol. (2008) 6:776–88. doi: 10.1038/nrmicro1978, PMID: 18794915 PMC2664199

[25] Qin J Li R Raes J Arumugam M Burgdorf KS Manichanh C. A human gut microbial gene catalogue established by metagenomic sequencing. Nature. (2010) 464:59–65. doi: 10.1038/nature08821, PMID: 20203603 PMC3779803

[26] Xie K Chang S Ning J Guo Y Zhang C Yan T. Dietary supplementation of Allium mongolicum modulates rumen-hindgut microbial community structure in Simmental calves. Front Microbiol. (2023) 14:14. doi: 10.3389/fmicb.2023.1174740, PMID: 37350783 PMC10284144

[27] Wang X Li F Zhang N Ungerfeld E Guo L Zhang X. Effects of supplementing a yeast culture in a pelleted total mixed ration on fiber degradation, fermentation parameters, and the bacterial community in the rumen of sheep. J Anim Sci Technol. (2023) 296:115565. doi: 10.1016/j.anifeedsci.2022.115565

[28] Cui X Wang Z Guo P Li F Chang S Yan T. Shift of feeding strategies from grazing to different forage feeds reshapes the rumen microbiota to improve the ability of Tibetan sheep (*Ovis aries*) to adapt to the cold season. Microbiol Spectr. (2023) 11:e0281622. doi: 10.1128/spectrum.02816-22, PMID: 36809032 PMC10100778

[29] Wei ZW Deng Sun BL Guo YQ. Effects of high-concentrate diet on gast rointestinal health of ruminants and its control measures. Chin J Anim Nutr. (2021) 33:1277–85. doi: 10.3969/j.issn.1006-267x.2021.03.010

[30] Qiao Y Sun J Xia S Tang X Shi Y le G. Effects of resveratrol on gut microbiota and fat storage in a mouse model with high-fat-induced obesity. Food Funct. (2014) 5:1241–9. doi: 10.1039/c3fo60630a, PMID: 24722352

[31] Ding W Liu WJ Ao CJ. Adding Allium mongolicum regel powder or compound probiotics to the diet rumen fermentation parameters and rumen fluid of Dorper × thin-tailed Han crossbred sheep the influence of bacterial diversity. Chin J Anim Nutr. (2019) 31:324–33. doi: 10.3969/j.issn.1006-267x.2019.01.039

[32] Gharechahi J Vahidi MF Bahram M Han JL Ding XZ Salekdeh GH. Metagenomic analysis reveals a dynamic microbiome with diversified adaptive functions to utilize high lignocellulosic forages in the cattle rumen. ISME J. (2021) 15:1108–20. doi: 10.1038/s41396-020-00837-2, PMID: 33262428 PMC8114923

[33] Bekele AZ Koike S Kobayashi Y. Genetic diversity and diet specificity of ruminal Prevotella revealed by 16S rRNA gene-based analysis. FEMS Microbiol Lett. (2010) 305:49–57. doi: 10.1111/j.1574-6968.2010.01911.x, PMID: 20158525

[34] Fernando SC Purvis HT II Najar FZ Sukharnikov LO Krehbiel CR Nagaraja TG. Rumen microbial population dynamics during adaptation to a high-grain diet. Appl Environ Microbiol. (2010) 76:7482–90. doi: 10.1128/AEM.00388-10, PMID: 20851965 PMC2976194

[35] Carberry CA Kenny DA Han S McCabe MS Waters SM. Effect of phenotypic residual feed intake and dietary forage content on the rumen microbial Community of Beef Cattle. Appl Environ Microbiol. (2012) 78:4949–58. doi: 10.1128/AEM.07759-11, PMID: 22562991 PMC3416373

[36] Ellison MJ Conant GC Lamberson WR Cockrum RR Austin KJ Rule DC. Diet and feed efficiency status affect rumen microbial profiles of sheep. Small Rumin Res. (2017) 156:12–9. doi: 10.1016/j.smallrumres.2017.08.009

[37] Su M Hao Z Shi H Li T Wang H Li Q. Metagenomic analysis revealed differences in composition and function between liquid-associated and solid-associated microorganisms of sheep rumen. Front Microbiol. (2022) 13:13. doi: 10.3389/fmicb.2022.851567, PMID: 35711780 PMC9197192

