# Supplementary material for: Effects of adding Allium mongolicum Regel powder and yeast cultures to diet on rumen microbial flora of Tibetan sheep (Ovis aries)
Source: Front Vet Sci. 2024 Feb 21;11:1283437. doi: 10.3389/fvets.2024.1283437 (PMC10914970; doi:10.3389/fvets.2024.1283437)
Supplement: Supplementary file 1 [file Table_1.docx]

Supplementary Material

## Supplementary Tables

**Supplementary Table 1** Sample sequencing data processing results statistics

| Sample ID | Raw CCS | Clean CCS | Effective CCS | AvgLen(bp) | Effective(%) |
| --- | --- | --- | --- | --- | --- |
| S-CON1 | 8,430 | 8,351 | 8,306 | 1,461 | 98.53 |
| S-CON2 | 8,621 | 8,578 | 8,362 | 1,461 | 97.0 |
| S-CON3 | 8,519 | 8,403 | 8,309 | 1,456 | 97.53 |
| S-CON4 | 7,963 | 7,876 | 7,348 | 1,460 | 92.28 |
| S-CON5 | 7,720 | 7,601 | 7,132 | 1,457 | 92.38 |
| S-CON6 | 6,909 | 6,843 | 6,499 | 1,459 | 94.07 |
| S-GroupⅠ1 | 6,982 | 6,966 | 6,721 | 1,456 | 96.26 |
| S-GroupⅠ2 | 8,431 | 8,392 | 8,298 | 1,459 | 98.42 |
| S-GroupⅠ3 | 6,882 | 6,860 | 6,638 | 1,462 | 96.45 |
| S-GroupⅠ4 | 8,098 | 8,062 | 7,847 | 1,458 | 96.9 |
| S-GroupⅠ5 | 7,553 | 7,533 | 7,387 | 1,458 | 97.8 |
| S-GroupⅠ6 | 8,049 | 8,044 | 7,961 | 1,461 | 98.91 |
| S-GroupⅡ1 | 7,762 | 7,742 | 7,463 | 1,455 | 96.15 |
| S-GroupⅡ2 | 7,306 | 7,280 | 7,125 | 1,457 | 97.52 |
| S-GroupⅡ3 | 7,706 | 7,602 | 7,162 | 1,457 | 92.94 |
| S-GroupⅡ4 | 7,847 | 7,828 | 7,408 | 1,455 | 94.41 |
| S-GroupⅡ5 | 8,176 | 8,155 | 7,985 | 1,457 | 97.66 |
| S-GroupⅡ6 | 8,605 | 8,537 | 8,207 | 1,459 | 95.37 |
| S-GroupⅢ1 | 7,955 | 7,788 | 7,674 | 1,456 | 96.47 |
| S-GroupⅢ2 | 7,323 | 7,313 | 7,194 | 1,464 | 98.24 |
| S-GroupⅢ3 | 7,009 | 6,916 | 6,568 | 1,457 | 93.71 |
| S-GroupⅢ4 | 8,352 | 8,204 | 8,141 | 1,457 | 97.47 |
| S-GroupⅢ5 | 7,040 | 6,937 | 6,844 | 1,456 | 97.22 |
| S-GroupⅢ6 | 8,623 | 8,545 | 8,487 | 1,457 | 98.42 |
| L-CON 1 | 8,054 | 7,957 | 7,883 | 1,458 | 97.88 |
| L-CON 2 | 7,669 | 7,643 | 7,459 | 1,459 | 97.26 |
| L-CON 3 | 7,316 | 6,997 | 6,810 | 1,454 | 93.08 |
| L-CON 4 | 7,027 | 6,865 | 6,507 | 1,459 | 92.6 |
| L-CON 5 | 7,859 | 7,583 | 7,254 | 1,456 | 92.3 |
| L-CON 6 | 8,334 | 8,034 | 7,778 | 1,455 | 93.33 |
| L-GroupⅠ1 | 8,249 | 8,179 | 7,499 | 1,463 | 90.91 |
| L-GroupⅠ2 | 7,582 | 7,549 | 6,918 | 1,467 | 91.24 |
| L-GroupⅠ3 | 8,076 | 8,035 | 7,653 | 1,461 | 94.76 |
| L-GroupⅠ4 | 6,930 | 6,912 | 6,343 | 1,466 | 91.53 |
| L-GroupⅠ5 | 8,113 | 8,021 | 7,455 | 1,459 | 91.89 |
| L-GroupⅠ6 | 8,158 | 8,127 | 7,581 | 1,464 | 92.93 |
| L-GroupⅡ1 | 8,049 | 8,042 | 7,429 | 1,461 | 92.3 |
| L-GroupⅡ2 | 7,495 | 7,417 | 6,987 | 1,460 | 93.22 |
| L-GroupⅡ3 | 8,184 | 7,825 | 7,519 | 1,464 | 91.87 |
| L-GroupⅡ4 | 7,576 | 7,507 | 7,092 | 1,460 | 93.61 |
| L-GroupⅡ5 | 7,061 | 6,921 | 6,356 | 1,460 | 90.02 |
| L-GroupⅡ6 | 6,760 | 6,663 | 6,169 | 1,461 | 91.26 |
| L-GroupⅢ1 | 7,138 | 6,854 | 6,407 | 1,455 | 89.76 |
| L-GroupⅢ2 | 7,634 | 7,611 | 7,483 | 1,461 | 98.02 |
| L-GroupⅢ3 | 7,008 | 6,957 | 6,644 | 1,455 | 94.81 |
| L-GroupⅢ4 | 7,797 | 7,580 | 6,642 | 1,457 | 85.19 |
| L-GroupⅢ5 | 7,506 | 7,212 | 6,817 | 1,456 | 90.82 |
| L-GroupⅢ6 | 7,412 | 7,316 | 6,982 | 1,457 | 94.2 |

Sample ID is the name of the sample; Raw-CCS is the number of CCS identified for the sample; Clean CCS is the number of sequences identified and removed from the primers; Effective-CCS is the number of sequences filtered for length and used for subsequent analyses after removal of chimeras; AvgLen (bp) is the average sequence length of the sample; Effective (%) is the percentage of Effective-CCS to Raw-CCS. Effective-CCS as a percentage of Raw-CCS.

**Supplementary Table 2** Statistical table of species of each grade of samples

| Sample | Kingdom | Phylum | Class | Order | Family | Genus | Species |
| --- | --- | --- | --- | --- | --- | --- | --- |
| S-CON1 | 1 | 13 | 18 | 31 | 52 | 106 | 125 |
| S-CON2 | 1 | 11 | 16 | 31 | 54 | 106 | 154 |
| S-CON3 | 1 | 10 | 16 | 27 | 47 | 106 | 137 |
| S-CON4 | 2 | 14 | 19 | 34 | 61 | 117 | 167 |
| S-CON5 | 2 | 12 | 18 | 33 | 55 | 113 | 140 |
| S-CON6 | 1 | 11 | 18 | 32 | 55 | 117 | 168 |
| S-GroupⅠ1 | 2 | 13 | 19 | 30 | 42 | 102 | 123 |
| S-GroupⅠ2 | 1 | 13 | 19 | 35 | 52 | 106 | 145 |
| S-GroupⅠ3 | 1 | 14 | 18 | 30 | 55 | 104 | 137 |
| S-GroupⅠ4 | 1 | 13 | 19 | 35 | 53 | 112 | 164 |
| S-GroupⅠ5 | 2 | 16 | 21 | 32 | 56 | 120 | 165 |
| S-GroupⅠ6 | 1 | 15 | 20 | 37 | 61 | 127 | 180 |
| S-GroupⅡ1 | 1 | 13 | 17 | 29 | 49 | 105 | 133 |
| S-GroupⅡ2 | 1 | 13 | 18 | 30 | 50 | 110 | 140 |
| S-GroupⅡ3 | 1 | 10 | 13 | 23 | 36 | 83 | 102 |
| S-GroupⅡ4 | 1 | 15 | 21 | 35 | 56 | 116 | 157 |
| S-GroupⅡ5 | 1 | 12 | 17 | 28 | 47 | 105 | 139 |
| S-GroupⅡ6 | 1 | 12 | 18 | 30 | 53 | 112 | 155 |
| S-GroupⅢ1 | 1 | 14 | 17 | 30 | 49 | 103 | 123 |
| S-GroupⅢ2 | 1 | 11 | 17 | 27 | 49 | 99 | 128 |
| S-GroupⅢ3 | 1 | 9 | 15 | 27 | 44 | 83 | 108 |
| S-GroupⅢ4 | 1 | 11 | 14 | 28 | 50 | 110 | 146 |
| S-GroupⅢ5 | 1 | 14 | 19 | 34 | 48 | 99 | 129 |
| S-GroupⅢ6 | 1 | 10 | 18 | 31 | 59 | 117 | 156 |
| L-CON 1 | 1 | 14 | 19 | 35 | 55 | 108 | 124 |
| L-CON 2 | 1 | 14 | 21 | 37 | 59 | 115 | 159 |
| L-CON 3 | 1 | 9 | 12 | 21 | 34 | 73 | 81 |
| L-CON 4 | 1 | 15 | 20 | 37 | 65 | 114 | 144 |
| L-CON 5 | 1 | 10 | 16 | 30 | 50 | 98 | 107 |
| L-CON 6 | 1 | 12 | 19 | 28 | 48 | 94 | 120 |
| L-GroupⅠ1 | 1 | 13 | 17 | 30 | 51 | 106 | 128 |
| L-GroupⅠ2 | 1 | 13 | 17 | 32 | 53 | 108 | 128 |
| L-GroupⅠ3 | 2 | 15 | 20 | 35 | 62 | 108 | 125 |
| L-GroupⅠ4 | 1 | 15 | 20 | 35 | 57 | 113 | 150 |
| L-GroupⅠ5 | 1 | 15 | 23 | 37 | 66 | 134 | 172 |
| L-GroupⅠ6 | 1 | 16 | 21 | 38 | 68 | 142 | 183 |
| L-GroupⅡ1 | 2 | 14 | 20 | 33 | 50 | 98 | 127 |
| L-GroupⅡ2 | 1 | 14 | 21 | 36 | 63 | 113 | 133 |
| L-GroupⅡ3 | 1 | 9 | 13 | 23 | 36 | 73 | 91 |
| L-GroupⅡ4 | 1 | 17 | 24 | 41 | 71 | 137 | 180 |
| L-GroupⅡ5 | 1 | 14 | 20 | 35 | 57 | 113 | 136 |
| L-GroupⅡ6 | 1 | 17 | 24 | 38 | 60 | 123 | 149 |
| L-GroupⅢ1 | 1 | 13 | 17 | 30 | 46 | 85 | 95 |
| L-GroupⅢ2 | 1 | 14 | 20 | 35 | 59 | 110 | 136 |
| L-GroupⅢ3 | 1 | 13 | 21 | 32 | 52 | 87 | 107 |
| L-GroupⅢ4 | 2 | 14 | 20 | 33 | 57 | 106 | 126 |
| L-GroupⅢ5 | 2 | 13 | 18 | 29 | 46 | 79 | 91 |
| L-GroupⅢ6 | 2 | 15 | 24 | 37 | 62 | 113 | 148 |
| Total | 2 | 19 | 32 | 55 | 108 | 209 | 341 |

.
